# Synthesis, Characterization, and Bactericidal Evaluation of Chitosan/Guanidine Functionalized Graphene Oxide Composites

**DOI:** 10.3390/molecules22010012

**Published:** 2016-12-23

**Authors:** Ping Li, Yangyang Gao, Zijia Sun, Dan Chang, Ge Gao, Alideertu Dong

**Affiliations:** 1 College of Chemistry, Jilin University, Changchun 130021, China; lping19@126.com (P.L.); liuzhenguang1234@163.com (Z.S.); changdan15@mails.jlu.edu.cn (D.C.); 2 College of Chemistry and Chemical Engineering, Inner Mongolia University, Hohhot 010021, China; tangyu2209@163.com

**Keywords:** graphene oxide, chitosan, polyhexamethylene guanidine hydrochloride, antibacterial activity

## Abstract

In response to the wide spread of microbial contamination induced by bacterial pathogens, the development of novel materials with excellent antibacterial activity is of great interest. In this study, novel antibacterial chitosan (CS) and polyhexamethylene guanidine hydrochloride (PHGC) dual-polymer-functionalized graphene oxide (GO) (GO-CS-PHGC) composites were designed and easily fabricated. The as-prepared materials were characterized by Fourier transform infrared (FTIR), X-ray photoelectron spectrometer (XPS), field emission scanning electron microscopy (FE-SEM), transmission electron microscope (TEM), thermogravimetric analysis (TGA) and Raman spectroscopy. Their antibacterial capability towards bacterial strains was also studied by incubating both Gram-negative bacteria and Gram-positive bacteria in their presence. More significantly, the synergistic antibacterial action of the three components was assayed, and the findings implied that the as-prepared GO-CS-PHGC shows enhanced antibacterial activity when compared to its single components (GO, CS, PHGC or CS-PHGC) and the mixture of individual components. Not only Gram-negative bacteria but also Gram-positive bacteria are greatly inhibited by GO-CS-PHGC composites. The minimum inhibitory concentration (MIC) value of GO-CS-PHGC against *E. coli* was 32 μg/mL. With the powerful antibacterial activity as well as its low cost and facile preparation, GO-CS-PHGC has potential applications as a novel antibacterial agent in a wide range of biomedical uses.

## 1. Introduction

The spread of antibiotic-resistant bacteria is a severe threat to public health worldwide [1]. There is great demand for a new generation of powerful antibacterial agents that can effectively kill pathogenic bacteria [2]. Nanomaterials, such as silver nanoparticles, carbon nanotubes, graphene-family nanomaterials and their composites, enable antibiotic-free disinfection of a broad spectrum of bacterial pathogens [3]. Graphene is a 2-dimensional (2D) sheet of sp^2^ hybridized carbon atoms which exhibits growing interesting prospects in various applications because of its extraordinary properties [4,5,6,7,8,9,10]. Graphene oxide (GO) obtained by chemical exfoliation of graphite, is decorated with a large number of oxygen moieties such as carboxyl, hydroxyl, and epoxy groups. These abundant oxygen functional groups of GO can provide reactive sites for chemical functionalization [11,12]. Moreover, GO has a 2D planar structure with a high specific area which can introduce interactions with the polymer matrix. Recent studies have demonstrated that GO is a biocompatible material, and has antibacterial ability with mild cytotoxicity [13,14,15,16,17,18,19,20,21,22,23]. The antibacterial activity of graphene and graphene derivatives is found to involve membrane puncture [24]. By direct contact, the sharp edges of graphene nanosheets can induce membrane stress, which results in physical damage to cell membranes, leading to the loss of bacterial membrane integrity and RNA leakage [24]. However, the present research results show that the antibacterial activity of GO is relatively low [3]. One potential path to realize practical antibacterial applications of GO is to potentiate its antibacterial activity.

The antibacterial activity of graphene-family materials and their hybrid materials have been widely studied in recent year [23,25,26,27,28,29,30,31,32,33,34,35,36,37]. Different antibacterial organic compounds and polymers were used to functionalize graphene-family materials in order to enhance the antibacterial activity of the resulting composites. Based on this strategy, CS, ramizol, benzalkonium bromide (BKB) and polyhexamethylene guanidine hydrochloride (PHGC) were all applied to obtain novel antibacterial agents with remarkable antibacterial activity [14,20,38,39,40]. Compared with low molecular organic compounds, antibacterial polymers typically show prolonged lifetimes, reduced residual toxicity and enhanced antibacterial activity. However, at present there are a few studies on functionalization of graphene-family materials with antibacterial polymers.

Guanidine polymer synthesized by the polycondensation of guanidinium and diamine has a wide spectrum of antibacterial activity, excellent bactericidal efficiency and non-toxicity [41,42]. However guanidine polymer-based antibacterial agents also have undeniable inherent defects. They are difficult to recycle owing to their good water-solubility, causing secondary contamination. If water soluble guanidine polymers are used as additives for industrial goods, the final products have low antibacterial fastness [43], so developing guanidine polymer composite materials with excellent antibacterial properties and strong antibacterial fastness under various conditions is still a critical need. Xiao et al. [40] synthesized guanidine-modified graphene with a high antibacterial activity using fluorinated graphene as a substrate. GO was observed to show the maximum antibacterial activity among a series of graphene-family materials (graphite, graphene and reduced graphene oxide) [44,45], so GO is the best option for the preparation of graphene-based antibacterial composites. Herein, we employed GO as a substrate to give new life to a common antibacterial agent-guanidine polymer. GO tends to aggregate in saline solution [46], and guanidine polymer is usually obtained as a salt, which causes the aggregation of GO. The aggregation of GO sheets significantly reduces the chances for interaction with bacteria for membrane puncture [44]. In our previous study, a polyethylene glycol (PEG) and PHGC dual-polymer-functionalized graphene oxide (GO-PEG-PHGC) antibacterial material was synthesized [47]. The result showed that PEG had a negligible antibacterial effect itself, but it enhanced the antibacterial activity of GO-PEG-PHGC. This better dispersion owing to the presence of PEG brings a greater contact between the bacterial membrane and nanomaterials, leading to greater bacterial membrane damage. If the PEG is substituted by another material which can achieve the same effect as PEG and possess a certain antibacterial activity, the novel composites will display much stronger bactericidal activity.

CS, a natural, low cost biomaterial, contains many active hydroxyl and amino functional groups on its backbone chain [48]. CS has been widely applied as a model polymer because of its excellent biocompatibility, biodegradability, antibacterial properties and adsorption capacity for contaminants [48,49,50,51,52]. One of the applications of CS that has attracted considerable interests is its role as an antibacterial agent. CS is highly soluble in acidic aqueous medium, but insoluble at alkaline solutions. CS existing as a disassociated form in solution with an extending conformation, which ensures sufficient contact with bacteria, resulting in better antibacterial activity. In an attempt to increase the antibacterial activity of CS over a wide pH range, the feasibility of chitosan/guanidine polymers was investigated by many researchers [53,54]. These studies suggest that the attachment of the guanidine group onto CS can introduce positive charges on the polymer backbone, which results in better aqueous solubility with enhanced antimicrobial activity at neutral pH. Xiao et al. [55] prepared chitosan-guanidine composites by reacting CS and PHGC. The results from their study showed that chitosan-guanidine composites demonstrate excellent antibacterial activity.

Blending of GO with CS used for antibacterial activity has been reported in recent years [56,57,58,59,60]. With multiple amino groups, CS has the potential to form composites with GO nanosheets. Epoxide, carboxyl, and hydroxyl groups decorated on the basal plane and edges of GO enable interactions with the functional groups of CS. Akhavan et al. [58] synthesized GO-CS composites with stacked layer structures and also applied them as antibacterial agents. The results illustrated that CS efficiently inhibited the growth of the bacteria. GO-CS composite layers consistently show significant antibacterial activity against *S. aureus*. However, there have been no reports about functionalization of GO with chitosan-guanidine composites to date. In this study, we report a new approach to potentiate the antibacterial activity of GO. CS-PHGC composites were bonded to the surface of GO sheets for the preparation of dual-polymer-functionalized graphene oxide (GO-CS-PHGC). The findings from this work provide a strategy for designing more efficient antibacterial graphene-based composites.

## 2. Results and Discussion

### 2.1. Characterization of GO-CS-PHGC Composites

#### 2.1.1. The Stability of GO-CS-PHGC Composites in Aqueous Solution

**Figure 1:**
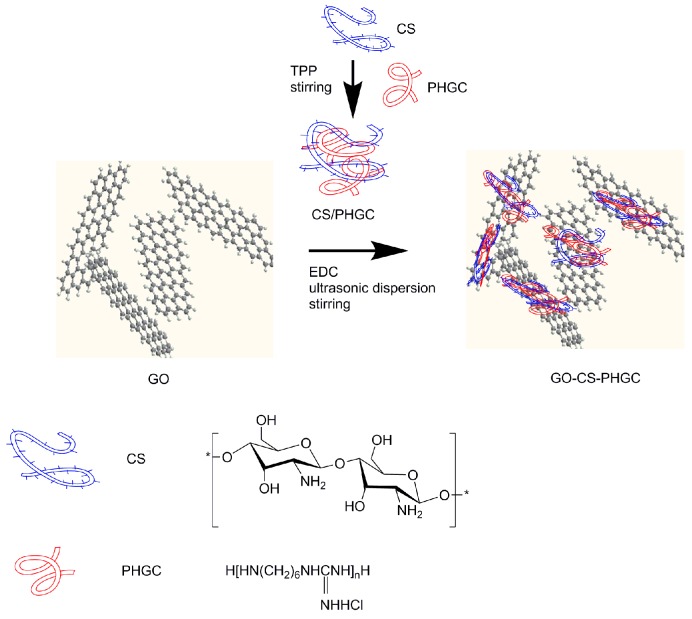
Synthetic route to GO-CS-PHGC.

**Figure 2:**
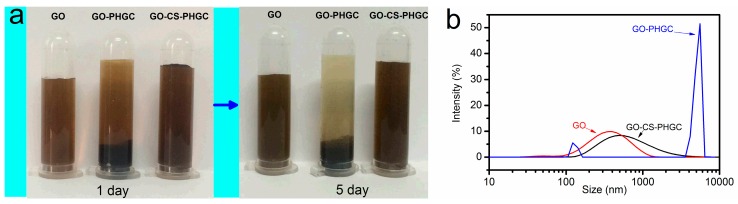
(**a**) Photographs of GO, GO-PHGC and GO-CS-PHGC dispersions after standing still for 1 day and 5 days in water (at the concentration 200 μg/mL); (**b**) Size and size distribution of GO, GO-PHGC and GO-CS-PHGC detected by DLS.

GO and GO-CS-PHGC were obtained as described in the Materials and Methods section. The preparation of GO-CS-PHGC composites is shown schematically in Figure 1. In order to verify the role of CS as stabilizer that give a better dispersion of GO-CS-PHGC, the stability of GO, GO-PHGC and GO-CS-PHGC were investigated by dispersing them in water. Photographs of GO, GO-PHGC and GO-CS-PHGC in aqueous solution (at a concentration of 200 μg·mL^−1^) are shown in Figure 2a. GO-PHGC was prepared according to our previous report [47]. The GO was able to disperse in aqueous solution after standing for several days due to the existence of a large amount of hydrophilic functional groups on the GO sheets. Most of GO-PHGC particles precipitated after the GO-PHGC dispersion stood for 1 day. This phenomenon is explained as follows: guanidine polymer is usually obtained as a salt, which causes the aggregation of GO. GO-CS-PHGC dispersion was brown in color and stable after standing for 5 days. This phenomenon is attributed to the existence of CS which prevents the composites from agglomerating. GO attaches large amount hydrophilic functional groups, such as carboxyl, hydroxyl, and epoxy groups. The hydrophilic functional groups of GO result in an individual sheet level dispersion of GO. As a hydrophilic biopolymer with -NH_2_ and -OH groups in each unit, CS favors the interaction with GO [61]. Hydrogen bonding and electrostatic attraction between GO and CS induce a truly homogeneous co-dispersion on the molecular scale and therefore, a good dispersion of CS-PHGC in GO sheets is expected. The size distribution was measured to evaluate the stability of GO-CS-PHGC in aqueous solution by dynamic light scattering (DLS), as shown in Figure 2b.

The average particle sizes of GO and GO-CS-PHGC are 405 nm and 613 nm, respectively, which indicates that the graft of CS-PHGC to the surface of GO does not significantly increase the particle size. The size of graphene-based materials can reflect their dispersion state in solution. GO and GO-CS-PHGC show equivalent diameters, which suggests GO-CS-PHGC and GO are stably distributed in aqueous solution. While the average particle size of GO-PHGC changes into 4693 nm, indicating significant the formation of agglomerates due to the interaction between GO and PHGC.

#### 2.1.2. FTIR Analysis

**Figure 3:**
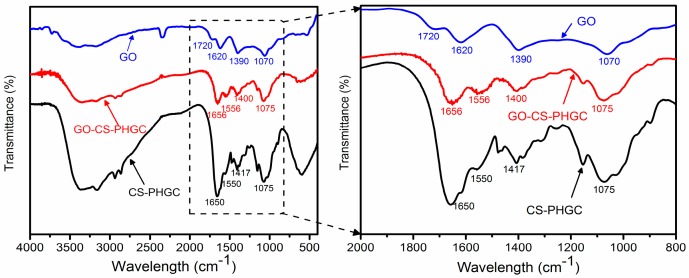
FTIR spectrum of GO, CS-PHGC and GO-CS-PHGC.

Direct evidence for the formation of GO-CS-PHGC is provided by FTIR spectroscopy as shown in Figure 3. The FTIR spectrum of GO is similar to the result previously reported in the literature [60]. Carboxyl groups (-COOH) are observed as bands at 1070 and 1720 cm^−1^. The band at ~1620 cm^−1^ is attributed to the presence of epoxy groups (C-O-C) and the C=C stretching mode of the sp^2^ carbon skeletal network. The band at 1070 cm^−1^ is also attributed to epoxy groups (C-O-C). The peak at 1390 cm^-1^ and the band between 2500 and 3800 cm^−1^ correspond to C-O-H stretching.

As can be seen in Figure 3, in comparison with GO, the FTIR spectrum of CS-PHGC and GO-CS-PHGC show an obvious peak appearing at 1650 cm^−1^, which is mainly attributed to the -NH stretching in CS molecules, confirming the presence of CS. For the FTIR spectrum of GO-CS-PHGC, the peak at 1650 cm^−1^ due to the stretching vibration of -NHCO- is shifted to 1656 cm^−1^, suggesting an interaction between CS-PHGC and GO. The FTIR spectra of GO-CS-PHGC and CS-PHGC exhibits an obvious C-N stretching vibration band at ∼1400–1410 cm^−1^ due to the existence of the secondary amine from PHGC. The results provide clear evidence for the successful conjugation of CS-PHGC and GO. Furthermore, the FTIR spectra of CS-PHGC and GO-CS-PHGC exhibit an obvious peak at ~1550 cm^−1^ due to N-H bending vibration of CS and PHGC.

#### 2.1.3. XPS Analysis

**Figure 4:**
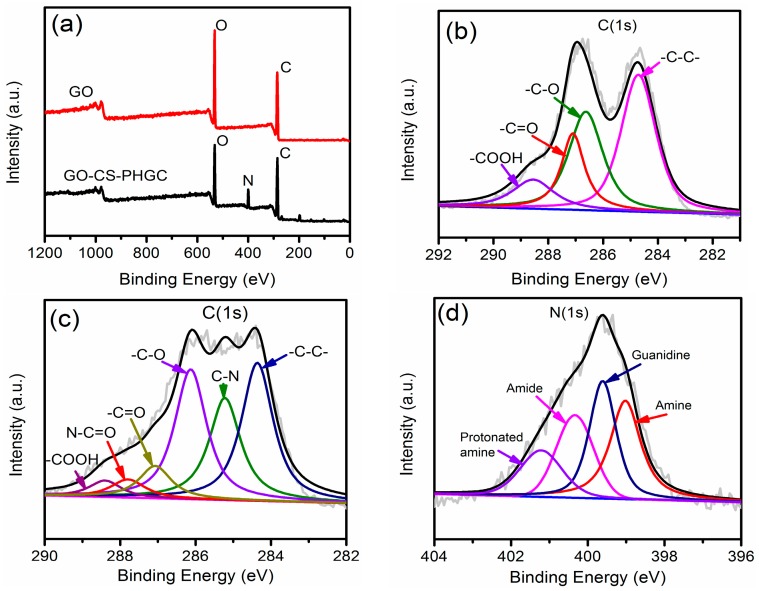
(**a**) XPS spectra of GO and GO-CS-PHGC; C 1s spectra of (**b**) GO and (**c**) GO-CS-PHGC with deconvoluted peaks; N 1s of (**d**) GO-CS-PHGC with deconvoluted peaks.

The XPS spectra of GO and GO-CS-PHGC were used to further analyze the chemical bonds of GO-CS-PHGC, which can be used as effective tools to characterize the presence of different elements such as carbon, nitrogen and oxygen. As shown in Figure 4a, a nitrogen signal cannot be observed in the GO curves, while nitrogen is found in GO-CS-PHGC, suggesting the successful incorporation of CS-PHGC. The C 1s curves of GO (Figure 4b) confirm the presence of a typical oxidized carbon that consisted of four fitting curves with binding energies of 284.8 (C-C), 286.4 (C-O), 287.1 (C=O), and 288.1 eV (O=C-O), respectively. It can be observed that two additional peaks for N-C=O at 287.8 eV and C-N bond at 285.2 eV in the C 1s curves of GO-CS-PHGC (Figure 4c), confirming the formation of amide linkages between CS-PHGC and GO. The similar results are also reported by other [62]. The broad peak of N 1s spectrum of GO-CS-PHGC (Figure 4d) can be fitted into four peaks at 401.2, 400.3, 399.6 and 399.0 eV, corresponding to the protonated amine, amide, guanidine (from PHGC) and amine, respectively.

#### 2.1.4. FE-SEM and TEM Analysis

**Figure 5:**
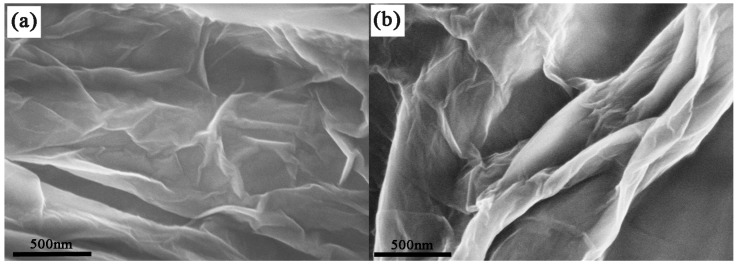

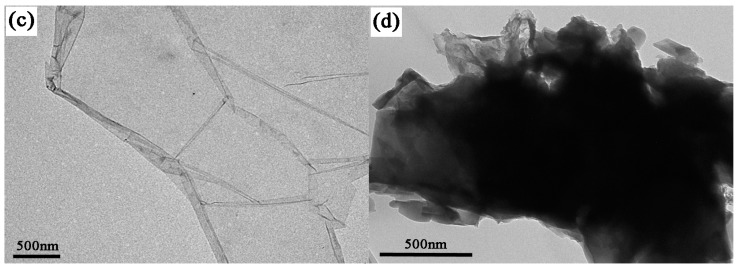
FE-SEM images of GO (**a**) and GO-CS-PHGC (**b**); TEM images of GO (**c**) and GO-CS-PHGC (**d**).

FE-SEM was adopted to visually examine the morphology of GO-CS-PHGC. The typical FE-SEM image of GO presents a wrinkled sheet-like structure, as shown in Figure 5a. Upon functionalization with CS-PHGC, the FE-SEM image of GO-CS-PHGC (Figure 5b) has a much rougher surface and greater thickness, which demonstrates that many polymer chains have been assembled on the surface of the GO sheets. TEM image of GO (Figure 5c) shows a transparent sheet with the presence of folded regions at the edges. TEM image of the GO-CS-PHGC (Figure 5d) presents a thick and rough sheet-like material, demonstrating that CS-PHGC composites have been assembled on the GO sheets.

#### 2.1.5. Raman Spectroscopy

**Figure 6:**
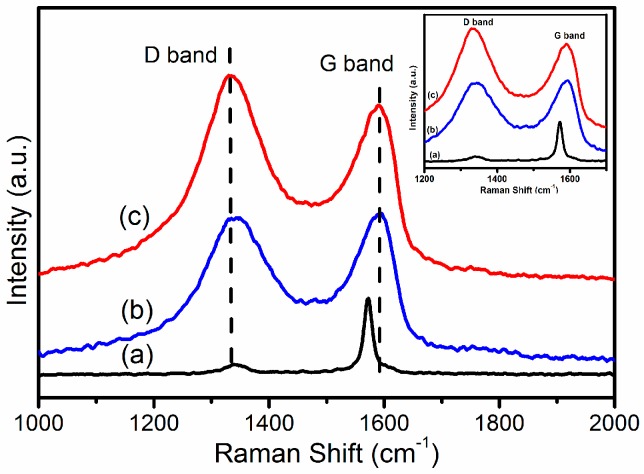
Raman spectra of (**a**) graphite, (**b**) GO and (**c**) GO-CS-PHGC.

Raman spectroscopy is known as an efficient method to examine the ordered/disordered crystal structures of carbon materials. The significant structural changes occurring during the modification process from graphite to GO-CS-PHGC have been identified by Raman spectra (Figure 6). Graphite shows a prominent G band at 1580 cm^−1^, which originates from the primary in-plane vibrational mode, and a quite weak D band at 1334 cm^−1^, indicating that there are some inherent defects in the graphite [11]. In the Raman spectra of GO, the G band and D band are located at 1595 cm^−1^ and 1350 cm^−1^, respectively. Compared with graphite, the D band of GO becomes more prominent, which is associated with the aromatic structure and the edge effect of GO. The G band of GO becomes broadened and shifts from 1580 cm^−1^ (graphite) to 1595 cm^−1^, which is ascribed to plane vibration of sp^2^ carbon atoms. Upon functionalization with CS-PHGC, the GO-CS-PHGC composites show two strong active Raman peaks at 1352 cm^−1^ (D band) and 1600 cm^−1^ (G band). The G band and D band have suffered a small red shift, caused by the defects in GO sheets formed during the chemical functionalization. Moreover, the intensity of D band becomes higher than that of G band. Generally, the intensity ratio of D and G bands (*I_D_/I_G_*) is the common metric that is used to characterize the defect density of graphene-based materials. For our samples, the *I_D_/I_G_* ratios of the GO and GO-CS-PHGC are 0.973 and 1.126, respectively, indicating the increase in structural distortion or defects after functionalization with CS-PHGC.

#### 2.1.6. TGA Analysis

**Figure 7:**
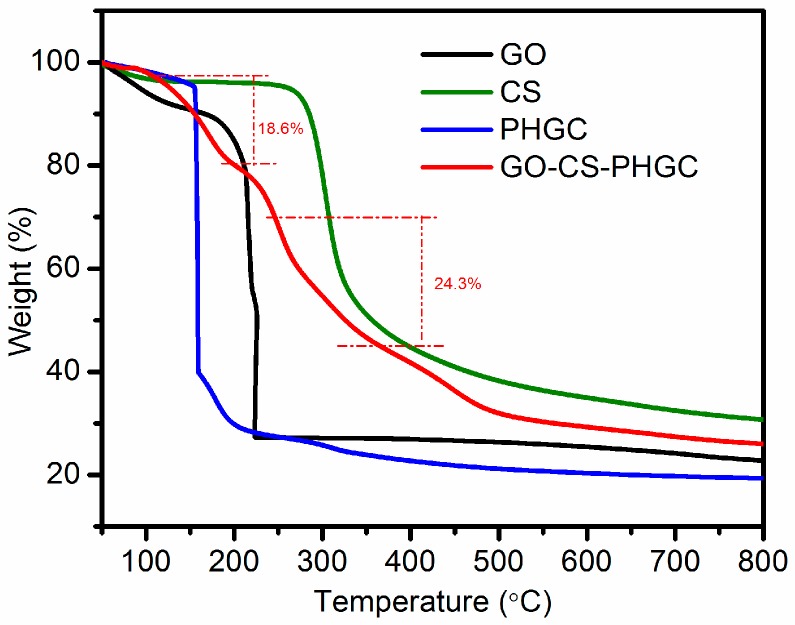
TGA curve of GO and GO-CS-PHGC.

TGA is a useful technique for determining the composition and thermal stability of materials. Figure 7 illustrates the TGA curves of the prepared materials. GO is thermally unstable as displayed in the TGA curves (Figure 7). Weight loss (12 wt %) of the GO up to 100 °C is primarily due to the evaporation of water molecules held in the samples. The major weight loss (72 wt %) around 210 °C is attributed to the decomposition of oxygen-containing groups. TGA data for CS and PHGC displays major weight losses around 290 °C and 155 °C for the decomposition of the polymer backbone, respectively. The TGA curve of GO-CS-PHGC demonstrates two main thermal events. The first one (18.6 wt %) around 155 °C is related to the decomposition of grafted PHGC chains, while the other one (24.3 wt %) around 290 °C is the decomposition of grafted CS chains. Weight loss is hardly found around 210 °C, which indicates that the main oxygen-containing groups of GO have been modified. The weight percentage of CS-PHGC on GO-CS-PHGC is 42.9 wt %, which is the summation of weight percentage of CS (24.3 wt %) and PHGC (18.6 wt %).

### 2.2. Antibacterial Test

#### 2.2.1. Biocidal Kinetic Test

**Figure 8:**
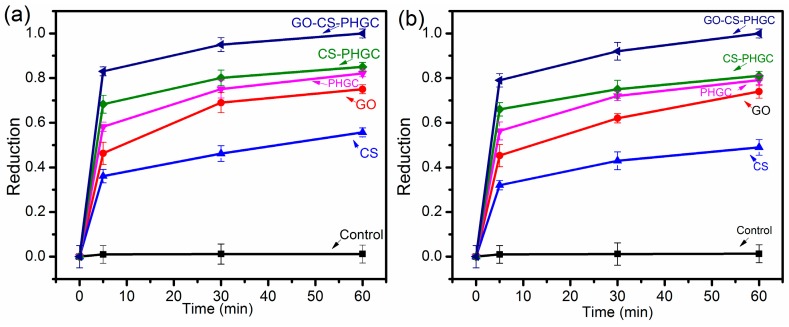

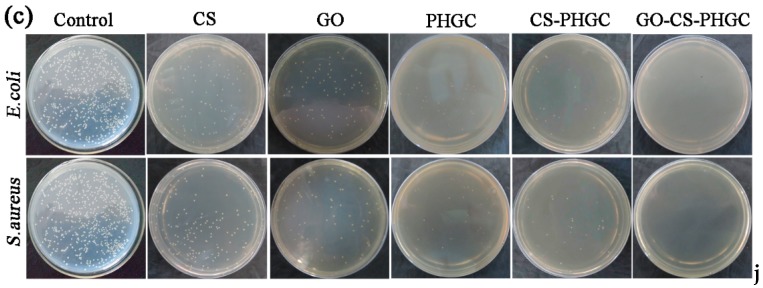
Reduction of bacterial colonies of (**a**) *E.* *coli* and (**b**) *S.* *aureus* after the exposure to Control, GO, CS, PHGC, CS-PHGC and GO-CS-PHGC composites. (The reduction = (*A* − *B*)/*A*; *A* is the number of surviving bacteria colonies on the control plate and *B* is that on the sample plate); (**c**) photographs of *E. coli* and *S. aureus* colonies grew on LB agar plates upon a 60 min exposure to Control, CS, GO, PHGC, CS-PHGC and GO-CS-PHGC.

The antibacterial activity test was performed for GO, CS, PHGC, CS-PHGC and GO-CS-PHGC using *E. coli* and *S. aureus* as model bacteria. The antibacterial activity of GO-CS-PHGC was investigated qualitatively by a biocidal kinetic test as shown in Figure 8a,b. As shown in Figure 8, CS-PHGC composites showed an improved antibacterial activity in comparison with PHGC and CS used alone. This result is similar to those of a previous study showing that the antibacterial activity of chitosan-guanidine composites is synergistically improved [55]. This result can be explained as follows: the rigidity of CS chains is enhanced with the introduction of guanidine polymer, and the CS chains are extended by electrostatic repulsion between the -NH^3+^ group of CS and cationic guanidio groups of PHGC. The PHGC chains associated with CS can contact bacteria through electrostatic attraction and destroy their membrane. The antibacterial efficiency of CS-PHGC composites is higher than that of CS and PHGC used alone. Similarly, the GO, CS, PHGC and CS-PHGC inactivation was measured for comparison to evaluate whether the functionalization enhanced the antibacterial efficiency of the prepared composites. To show the antibacterial activity directly, photographs of the antibacterial effects were obtained by culturing each sample with the same volume on LB agar plate exposed to the bacteria.

Figure 8c displays the bacterial survival after a 60 min exposure to GO, CS, PHGC, CS-PHGC and GO-CS-PHGC. The small white dots on the culture plates represent the bacterial colonies of *E. coli* and *S. aureus.* Of note, there are still some surviving bacterial colonies in the GO, CS, PHGC and CS-PHGC, while there are no remaining colonies in GO-CS-PHGC. GO-CS-PHGC composites indicating a complete kill of *E. coli* and *S. aureus* in less than 60 min. CS, GO, PHGC and CS-PHGC composites achieve 55%, 75%, 82% and 85% *E. coli* reduction in a contact time of 60 min, versus 49%, 73%, 79% and 81% for *S. aureus* reduction, respectively. This result demonstrates that the GO-CS-PHGC composites possess remarkable growth inhibition effects towards *E. coli* and *S. aureus*. In a previous report, the antibacterial activity of GO-PEG-PHGC was been studied, and the number of the bacterial colonies (*E. coli*) on GO-PEG-PHGC decreased 74.4% after 30 min of incubation [47]. Akhavan et al. [58] reported the antibacterial activity of GO-CS composites against *S. aureus*. The result showed more than 77% inactivation after 3 h. Wang et al. [63] synthesized an antibacterial membrane by fabricating a graphene oxide-silver NPs (GO-Ag) composite onto a cellulose acetate membrane. The GO-Ag based antibiofouling membrane exhibited a strong antibacterial activity, leading to an 86% inactivation of *E. coli* after contact with the membrane for 2 h. In this study, GO-CS-PHGC composites provide 95.0% *E. coli* and 92.0% *S. aureus* reduction after 30 min incubation. The remarkable enhanced antibacterial activity of GO-CS-PHGC composites may be related to a synergistic expression of individual components. This question will be further investigated in the following study.

#### 2.2.2. MIC Study

The experimental results mentioned above indicate that GO-CS-PHGC composites show higher antibacterial efficiency than that of any single component (GO, CS, PHGC and CS-PHGC). We wondered whether the remarkable enhancement of antibacterial activity is a simple additive effect of the three components GO, CS and PHGC or if it is an expression of a synergistic antibacterial effect of the individual components. To address this question, the MIC values of GO-CS-PHGC, the mixture of GO, CS and PHGC (GO + CS + PHGC) and the mixture of GO and CS-PHGC (GO + (CS-PHGC) were determined by using *E. coli* as model bacterium. The ratio of each component for GO + CS + PHGC and GO + (CS-PHGC) is the same as in the GO-CS-PHGC composites. Table 1 presents the MIC values obtained for the antibacterial agents. As shown, GO + CS + PHGC (MIC 256 μg/mL) and GO + (CS-PHGC) (MIC 128 μg/mL) exhibit a weaker antibacterial activity compared with GO-CS-PHGC (MIC 32 μg/mL). Therefore the significant antibacterial activity of GO-CS-PHGC composites is not due to a simple addition of contributions from individual components, but rather due to the unique physical and chemical characteristics of all components which contribute together in a synergistic manner to reveal a novel, remarkable efficient antibacterial activity.

The antibacterial activity of graphene oxide is attributed to membrane puncture [24,44]. The mechanism for the antibacterial effect of PHGC is analogous to that of GO due to the destruction of the bacterial membrane. Guanidine polymer inhibits bacterial growth by attacking them through electrostatic attraction between the cationic guanidino groups and anionic groups on the cell surface of bacteria. After attaching to bacteria cells, guanidine polymer induces bacterial membrane collapse and the intracellular components leak thereafter [64]. Similarly, the antibacterial mechanism of CS is based on the damaging interaction of the polycation (protonated amino groups) with the negatively charged surfaces of bacteria, resulting in loss of membrane permeability, cell leakage, and finally cell death [65]. As proposed in previous studies [36], GO sheets with more sharp edges have a stronger interaction with the cell membrane of the bacteria, which finally results in deeper damage of the cell membrane of bacteria during the contact interaction. Thus, the aggregation of GO-based materials inevitably reduces the chances of damaging the bacterial cell. Herein, CS, acting as both stabilizer and antibacterial agent, prevents the aggregation of GO-CS-PHGC and kills the bacteria. As a result, the synergistic effect can be explained as follows: (i) GO sheets with large specific surface area are proposed as an ideal nanostructure, which can increase the contact between CS-PHGC decorated on GO and the bacteria; (ii) the antibacterial activity of CS-PHGC composites is improved in comparison with PHGC and CS used alone due to the synergistic effect of CS and PHGC; (iii) the enhanced antibacterial activity is also described to be related to a better dispersion of GO-CS-PHGC in the presence of CS. The better dispersion allows the enhancement of direct contact between bacteria and materials, and therefore leading to larger cell damage. As regards to above considerations, the novel GO-CS-PHGC composites, combining the synergistic expression of the antibacterial activity of individual components, display remarkable antibacterial activity.

## 3. Materials and Methods

### 3.1. Materials

Nature graphite powder was purchased from Tianjin Guangfu Fine Chemical Research Institute (Tianjin, China). CS (degree of deacetylation (DD) = 95%), guanidine hydrochloride and hexamethylenediamine was purchased from Sinopharm Chemical Reagent Co., Ltd. (Shanghai, China). Concentrated H_2_SO_4,_ sodium nitrate potassium permanganate and H_2_O_2_ (30%) were obtained from Beijing Chemical Company (Beijing, China). 1-(3-Dimethylaminopropyl)-3-ehylcarbodiimide hydrochloride (EDC) was purchased from Sigma-Aldrich (Steinheiro, Germany). Sodium tripolyphosphate (TPP) was obtained from Aladdin Industrial Corporation (Shanghai, China).

### 3.2. Preparation of GO

Crude graphite oxide was obtained by the modified Hummers method [66]. Commercial graphite powder (1.0 g) was stirred in cold (0 °C) concentrated sulfuric acid (23 mL) and then sodium nitrate (1.2 g) was added to the suspension. Potassium permanganate (3.0 g) was added gradually with stirring and cooling, the temperature of the mixture was controlled below 20 °C. The mixture was stirred at 35 °C for 4 h. After that, distilled water (40 mL) was slowly added to the mixture with the temperature maintained below 98 °C. The dilute suspension was stirred at 95 °C for an additional 15 min. The reaction was terminated by the addition of a large amount of distilled water (100 mL), and then 30% H_2_O_2_ solution (3.0 mL) was added. The mixture was left 1 h. The GO particles settled at the bottom were separated from the excess liquid by decantation. The remaining suspension was transferred to dialysis tubes. Dialysis was carried out until no precipitate of BaSO_4_ was detected by addition of an aqueous solution of BaCl_2_. Finally the crude graphite oxide was obtained. GO sheets were exfoliated from crude graphite oxide by sonication.

### 3.3. Preparation of PHGC

PHGC was synthesized by melt polymerization. Equimolar amounts of guanidine hydrochloride and hexamethylenediamine were added to a flask. The mixture was heated to 120 °C and stirred for 2 h. Then the mixture was heated to 160 °C and stirred for 6 h. During this period, the evolving NH_3_ started to evolve until no any NH_3_. After the reaction, PHGC was obtained.

### 3.4. Preparation of Chitosan-Guanidine Composites

Chitosan-guanidine composites were obtained according to the synthetic procedure given in the literature [55]. Chitosan (0.20 g) was dissolved in 2.0% (*w*/*v*) acetic acid solution and 0.20 g of PHGC was added and stirred for 30 min. 1.0 mL of TPP (initial concentration 0.1 mol·L^−1^) was added as a cross linking agent, and stirred for 4 h at room temperature.

### 3.5. Preparation of GO-CS-PHGC

GO (0.10 g) dispersion was prepared by sonicating GO for 30 min in ultrapure water. A solution of EDC (0.05 M) was added to the GO dispersion with continuous stirring for 2 h in order to activate the carboxyl groups of GO. CS-PHGC (0.10 g) and the activated GO solution were added in a flask and dispersed in distilled water by ultrasonic dispersion for 10 min. After ultrasonic dispersion, the mixed solutions were stirred at 60 °C for 2 h. The homogeneous GO-CS-PHGC solutions were poured into dialysis tubes for 7 day.

### 3.6. Characterization

FTIR of the composites was obtained on a FTIR-8400 spectrometer (Shimadzu, Kyoto, Japan) in the range of 4000–400 cm^−1^ and averaged over 32 scans at room temperature. The samples were prepared using powder pressed KBr pellets. FE-SEM was observed with a SU8020 field emission scanning electron microscope (Hitachi, Tokyo, Japan) at a voltage of 3 kV. TEM images were taken on a Hitachi H-8100 transmission electron microscope at 8 kV. TGA was performed with a PerkinElmer (Norwolk, CT, USA) thermogravimetric analyzer from 50 °C to 800 °C under a N_2_ atmosphere (heating rate: 10 °C/min). The chemical composition of GO and GO-CS-PHGC was evaluated by XPS (ESCALAB 250, Thermo, Woburn, MA, USA) using 150 W monochromatic Al Kα radiation. Raman spectroscopy (LabRAM HR Evolution, Horiba, Kyoto, Japan) of the graphite, GO, and GO-CS-PHGC particles was performed using a 514 nm wavelength laser as optical source for excitation. The particle size distributions were tested by DLS (Nano ZS90, Malvern, Worcestershire, UK), the samples were dispersed in water at a concentration of 200 μg/mL (based on GO).

### 3.7. Bacterial Culture and Antibacterial Test

*E. coli* and *S. aureus* were added to fresh LB medium for incubation overnight at 37 °C, and harvested in the mid-log phase. Cultures were centrifuged at 6500 rpm for 15 min. Pellet cells were obtained and washed twice with phosphate-buffered saline to remove residual macromolecules and other growth medium constituents. The bacterial suspensions employed for the tests contained 5 × 10^5^ CFU/mL. LB medium and phosphate-buffered saline were obtained according to the standard procedure. Disinfection kinetic test was evaluated with colony counting method.

As for antibacterial kinetic test, each sample was dispersed in sterilized DI water (2.0 mg/mL) under sonication for 15 min. Every sample solution (450 μL) was mixed with the bacteria suspension (50 μL), and then was incubated on a rotary shaker with the rotation speed of 170 rpm at 37 °C. After a certain period of contact time, the reaction suspensions were serially diluted, and then 100 μL of each dilution was dispersed into LB agar plates. The LB agar plates were incubated in an incubator at 37 °C for 24 h. Then colonies on the LB agar plates were counted and recorded. Sterilized distilled water without sample in the same way of sample testing (blank solution) was employed as negative control. The as-prepared mixture was dispersed onto LB agar plates after serial dilution to incubate at 37 °C for 24 h. All tests were repeated at least three times.

Samples with different serial concentrations were used to determine the MIC by standardized agar dilution. Briefly, Log phase *E. coli* (5 × 10^5^ CFU/mL) were spotted on a series of agar plates containing samples with different serial concentrations and incubated overnight at 37 °C. The MIC values are defined as the lowest concentration of antibacterial agent at which there was no visible bacterial growth.

## 4. Conclusions

In summary, dual-polymer-functionalized graphene oxide (GO-CS-PHGC) was prepared by conjugating CS-PHGC complexes onto the surface of GO sheets. CS-PHGC composites were employed to potentiate the antibacterial activity of GO. GO-CS-PHGC demonstrates superior antibacterial properties against both Gram negative bacteria (*E. coli*) and Gram positive bacteria (*S. aureus*). The high inactivation of GO-CS-PHGC is related to the synergistic expression of the antibacterial activity of the individual components. Furthermore, PHGC displays strong antibacterial fastness under various conditions. The novel GO-CS-PHGC composite material reported in this study as, can be used as a cost-effective disinfection agent for different applications to effectively inhibit bacterial growth and propagation.

## Figures and Tables

**Table 1:** MIC values of GO-CS-PHGC, the mixture of GO, CS and PHGC (GO + CS + PHGC) and the mixture of GO and CS-PHGC (GO + (CS-PHGC)) against *E. coli*.

Sample	GO-CS-PHGC	GO + (CS-PHGC)	GO + CS + PHGC
MIC (μg/mL)	32	128	256
